# Preliminary Phytochemical Screening, In Vitro Antidiabetic, Antioxidant Activities, and Toxicity of Leaf Extracts of *Psychotria malayana* Jack

**DOI:** 10.3390/plants10122688

**Published:** 2021-12-07

**Authors:** Tanzina Sharmin Nipun, Alfi Khatib, Qamar Uddin Ahmed, Mohd Hamzah Mohd Nasir, Farahaniza Supandi, Muhammad Taher, Mohd Zuwairi Saiman

**Affiliations:** 1 Pharmacognosy Research Group, Department of Pharmaceutical Chemistry, Kulliyyah of Pharmacy, International Islamic University Malaysia, Kuantan 25200, Pahang Darul Makmur, Malaysia; tsn.np99@gmail.com (T.S.N.); quahmed@iium.edu.my (Q.U.A.); mtaher@iium.edu.my (M.T.); 2 Department of Pharmacy, Faculty of Biological Sciences, University of Chittagong, Chittagong 4331, Bangladesh; 3 Faculty of Pharmacy, Airlangga University, Surabaya 60155, Indonesia; 4 Central Research and Animal Facility, Kulliyyah of Science, International Islamic University Malaysia, Kuantan 25200, Pahang Darul Makmur, Malaysia; hamzahn@iium.edu.my; 5 Institute of Biological Sciences, Faculty of Science, University of Malaya, Kuala Lumpur 50603, Malaysia; farahaniza@um.edu.my; 6 Centre for Research in Biotechnology for Agriculture (CEBAR), University of Malaya, Kuala Lumpur 50603, Malaysia; 7 Centre for Natural Products Research and Drug Discovery (CENAR), University of Malaya, Kuala Lumpur 50603, Malaysia

**Keywords:** *Psychotria malayana* Jack, antidiabetic activity, antioxidant activity, toxicity, GC-MS, derivatization

## Abstract

*Psychotria malayana* Jack belongs to the Rubiacea and is widespread in Southeast Asian countries. It is traditionally used to treat diabetes. Despite its potential medicinal use, scientific proof of this pharmacological action and the toxic effect of this plant are still lacking. Hence, this study aimed to investigate the in vitro antidiabetic and antioxidant activities, toxicity, and preliminary phytochemical screening of *P. malayana* leaf extracts by gas chromatography-mass spectrometry (GC-MS) after derivatization. The antidiabetic activities of different extracts of this plant were investigated through alpha-glucosidase inhibitory (AGI) and 2-NBDG glucose uptake using 3T3-L1 cell line assays, while the antioxidant activity was evaluated using DPPH and FRAP assays. Its toxicological effect was investigated using the zebrafish embryo/larvae (*Danio rerio*) model. The mortality, hatchability, tail-detachment, yolk size, eye size, beat per minute (BPM), and body length were taken into account to observe the teratogenicity in all zebrafish embryos exposed to methanol extract. The LC_50_ was determined using probit analysis. The methanol extract showed the AGI activity (IC_50_ = 2.71 ± 0.11 μg/mL), insulin-sensitizing activity (at a concentration of 5 µg/mL), and potent antioxidant activities (IC_50_ = 10.85 μg/mL and 72.53 mg AAE/g for DPPH and FRAP activity, respectively). Similarly, the water extract exhibited AGI activity (IC_50_ = 6.75 μg/mL), insulin-sensitizing activity at the concentration of 10 μg/mL, and antioxidant activities (IC_50_ = 27.12 and 33.71 μg/mL for DPPH and FRAP activity, respectively). The methanol and water extracts exhibited the LC_50_ value higher than their therapeutic concentration, i.e., 37.50 and 252.45 µg/mL, respectively. These results indicate that both water and methanol extracts are safe and potentially an antidiabetic agent, but the former is preferable since its therapeutic index (LC_50_/therapeutic concentration) is much higher than for methanol extracts. Analysis using GC-MS on derivatized methanol and water extracts of *P. malayana* leaves detected partial information on some constituents including palmitic acid, 1,3,5-benzenetriol, 1-monopalmitin, beta-tocopherol, 24-epicampesterol, alpha-tocopherol, and stigmast-5-ene, that could be a potential target to further investigate the antidiabetic properties of the plant. Nevertheless, isolation and identification of the bioactive compounds are required to confirm their antidiabetic activity and toxicity.

## 1. Introduction

Diabetes mellitus (DM) is one of the most persistent metabolic diseases. It is a disorder in which blood sugar levels rise, disrupting the body’s regular carbohydrate, lipid, and protein metabolism and eventually leading to death if not adequately treated or controlled [1]. Global diabetes prevalence is anticipated to reach 578 million (10.2 percent) in 2030, rising to 700 million (10.9 percent) by 2045 [2]. It is necessary to manage hyperglycemia because it may lead to severe complications. It can be controlled by keeping the blood glucose levels steady within the normal range (<7.8 mmol/L or <140 mg/dL). One of the potential approaches to control blood sugar level is by consuming medicinal herbs, which have been used traditionally to treat the disease. The screening of the antidiabetic agent from plants with minimal side effects has therefore attracted growing attention.

*Psychotria malayana* is a plant commonly available in Southeast Asian regions and is traditionally used to manage diabetes. The height of this plant is roughly about 1–4 m, and the Lombok people in Indonesia have utilized the water extract of this plant to prevent and treat some illnesses, including diabetes [3,4]. Despite its long history of traditional use as a therapeutic herb, the research on this plant’s glucose uptake and antioxidant activity is limited. This plant has been reported to possess alpha-glucosidase inhibitory activity, a key enzyme corresponding to type 2 diabetes [5,6]. In addition, the water extract of this plant reported to have the antidiabetic activity on the type 1 diabetic adult zebrafish model [7]. However, the report described only a polar extract of this plant, regardless of the possibility of finding the bioactivity on the broader polarity spectrum. No toxicological studies have ever been reported on this plant until now.

The majority of type 2 diabetic (T2DM) patients suffer from severe and extreme insulin resistance. Several studies focused on adipose tissue as a potential key mediator of whole-body insulin resistance [8]. The 3T3-L1 cell was formed by the clonal expansion of Mouse Swiss 3T3 cells. It is the most frequently used cell line of preadipocytes obtained from disaggregated Swiss 3T3 mouse embryos at 17–19 days old [9]. Plenty of research has been conducted on adipocytes’ adipogenesis and biochemistry using this cell line [10]. Adipocytes play a crucial function in the metabolism of glucose. After differentiation into adipocytes, 3T3-L1 preadipocytes serve as effective in vitro models that are useful for assessing glucose metabolism [11]. A new class of antidiabetic medications, thiazolidinedones (TZDs) like rosiglitazone, troglitazone, and pioglitazone, has been synthesized to treat patients suffering from T2DM, which make fat cells more responsive to insulin. TZDs enhance insulin sensitivity by stimulating the peroxisome proliferator-activated receptor-gamma (PPAR-γ). However, TZDs have been linked to causing detrimental cardiovascular side effects and may also cause bladder cancer [12]. Hence, this study discovers and examines the ability of *P. malayana* to improve insulin sensitivity.

The inhibition of carbolytic enzymes, such as alpha-glucosidase (AG), is one of the approaches for treating T2DM [13]. AG inhibitors can significantly regulate the post-prandial blood glucose levels by slowing the breakdown of carbohydrates and minimizing monosaccharides absorption [14]. Therefore, the discovery of new AG inhibitors is of critical interest for the management of T2DM.

Besides AG inhibitors and insulin sensitizers, antioxidants also play a vital role in the management of diabetes. Various chronic health issues, including diabetes, cancer, central nervous system disorders, and atherosclerosis, may arise from free radicals. In addition to scavenging free radicals, antioxidants have also been shown to inhibit free-radical reactions by contributing hydrogen atoms and quenching singlet oxygen [15,16]. Generally, more than one approach should be taken into consideration to investigate the antioxidant activity of plant-based extracts or compounds under investigation [15]. Therefore, in this study, two methods (2,2-diphenyl-1-picrylhydrazyl radical scavenging assay, i.e., DPPH and Ferric Reducing Antioxidant Power [FRAP] assays) were used to evaluate the antioxidant properties of *P. malayana* leaf extracts.

Inadequate safety data of medicinal herbs may cause severe toxic effects, like neurotoxicity, cardiotoxicity, hepatotoxicity, and nephrotoxicity [17]. For this reason, a toxicity study is very important to identify the possible risks associated with the human consumption of medicinal plants [18]. The objective of toxicity testing is to determine how safe a drug is and to identify the potentially toxic effects it can cause [19]. The determination of LC_50_ is the primary step to evaluate the toxic characteristics of a drug. Data from the acute toxicity test help to determine LC_50_ through which the therapeutic index of a new drug can be obtained. A new compound or drug is considered safe if its therapeutic index is high [20].

The use of embryonic stages of zebrafish (*Danio rerio*) is increasing since they are considered a replacement model for biomedical research and toxicology and allow the analysis of multiple endpoints ranging from acute to developmental toxicity [21]. Fish embryos are considered replacement or refinement models because their developmental stages are likely to experience less or no pain, suffering, or lasting harm [22]. In particular, the zebrafish has become an inevitable animal model for evaluating drug safety and toxicity [23]. Its embryo has achieved some practical virtues, including tiny size, growth outside the mother, and inexpensive maintenance of the adults, making it an excellent model and attractive to the toxicologist [24]. In 2005, the German Federal Environment Agency replaced adult fish used for the traditional toxicological tests with zebrafish embryos for the wastewater treatment test. It introduced the acute toxicity test as a standardized ISO assay [25,26]. Moreover, the Organization for Economic Co-operation and Development (OECD) issued guidelines for toxicity assessment. These guidelines outline a Fish Embryo Acute Toxicity Test using *D. rerio* embryos and designed to determine the acute chemical toxicity at embryonic fish stages [27].

Based on the above research gap, the present study aimed to evaluate in vitro the antidiabetic and antioxidant activities, toxicity, as well as preliminary phytochemical screening of *P. malayana* leaf extracts by GC-MS after derivatization.

## 2. Results

### 2.1. Yield of Extraction of P. malayana Leaves

The percentage yields of *P. malayana* leaves with *n*-hexane, ethyl acetate, methanol, and water are shown in Table 1. From the findings, it was observed that the percentage of extraction yield of methanol (31.51%) was significantly higher (*p* < 0.05) compared to water (20.40%), ethyl acetate (18.87%), and *n*-hexane (1.06%). The latter produced a significantly lower yield of extraction compared to the other three solvents (*p* < 0.05). On the other hand, there was no notable difference in the extraction yield between ethyl acetate and water.

### 2.2. AGI Activity of P. malayana Leaf Extracts

The AGI activity of ethyl acetate, methanol, and water extracts of *P. malayana* leaf is shown in Table 1. The AGI activity of n-hexane extract was not evaluated because it was found to be insoluble in DMSO. From this preliminary study, it was observed that the highest inhibition effect (IC_50_ = 2.71 μg/mL) against alpha-glucosidase enzyme was shown by methanol extract compared to ethyl acetate (IC_50_ = 5.37 μg/mL) and water (IC_50_ = 6.75 μg/mL). The findings indicate that all extracts (except *n*-hexane) displayed potent AGI activity.

In the present study, only methanol and water extracts were considered for further studies (2-NBDG glucose uptake, antioxidant, and GC-MS analysis after derivatization) because the methanol extract showed the highest activity in the AGI assay while water is considered the safest, cheapest, and abundantly available solvent for extraction. Besides, the AGI activity of the water extract was also superior and comparable to the methanol extract.

### 2.3. 2-NBDG Uptake in 3T3-L1 Cell Assay

#### 2.3.1. Cell Viability

**Figure 1:**
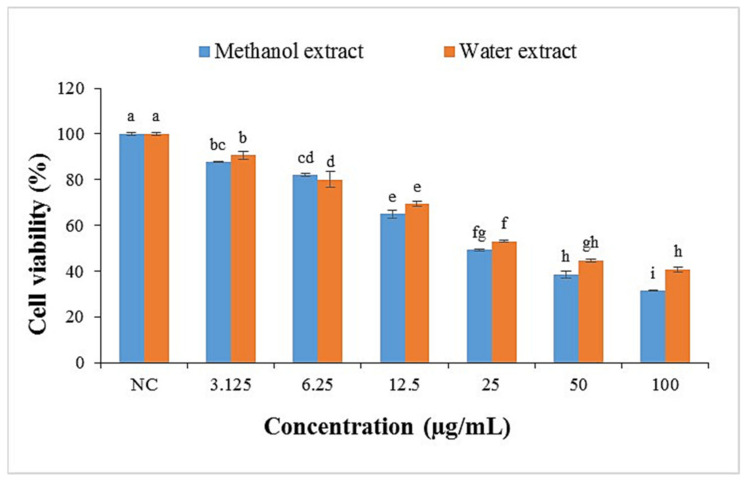
The cell viability (%) of 3T3-L1 preadipocyte cells measured by the MTT assay treated with methanol and water extracts of *P. malayana* leaves at the concentration of 3.125 to 100 μg/mL for 24 h. Data with a different letter are significantly different with the a *p*-value less than 0.05. Tukey’s multiple comparison test was used to calculate the data with a 95% confidence level; *n* = 3. NC: Negative control.

In the present study, crude extracts were treated on 3T3-L1 preadipocyte cells using the MTT assay to determine the safe concentration of the plant extracts in six sample concentrations (3.125, 6.25, 12.5, 25, 50, and 100 μg/mL). It was found that the viability of the cells treated with either *P. malayana* leaf methanol or water extracts showed notable differences (*p* < 0.05) in comparison to that of the untreated cells at all studied concentrations (Figure 1). Moreover, both extracts exhibited a dose-dependent effect. The viability of the cells treated with the methanol extract increased significantly as the sample concentration decreased up to 6.25 μg/mL. However, the viability of the cells treated with the water extract decreased significantly as the concentration increased up to 50 μg/mL. Moreover, there was no significant difference regarding the cell viability in methanol and water extracts at all studied concentrations except 100 μg/mL. The IC_50_ values of water and methanol were 35.46 and 30.53 μg/mL, respectively, and the concentration of treatments below IC_50_ was considered safe for further study.

#### 2.3.2. 2-NBDG Uptake in 3T3-L1 Cells

**Figure 2:**
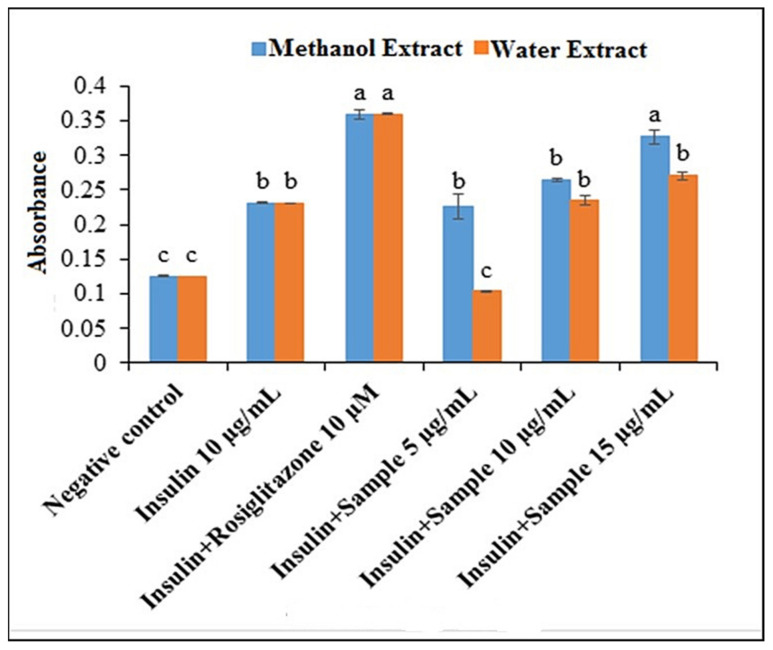
The effect of methanol and water extracts on glucose [2-NBDG] uptake measurement of 3T3-L1 adipocyte cells. Data with a different letter are significantly different with a *p*-value less than 0.05. Tukey’s multiple comparison test was used to calculate the data with a 95% confidence level; *n* = 3.

The effect of both extracts on the glucose uptake determination of 3T3-L1 adipocyte cells is shown in Figure 2. The glucose uptake by the untreated adipocytes was the lowest. Compared to untreated cells, insulin-treated cells took up significantly higher amounts of glucose. On the other hand, the cells treated with rosiglitazone in the presence of insulin were reported to have significantly higher uptake than the negative control and insulin groups, with absorbance values of 0.360, 0.126, and 0.232, respectively. Both extracts (methanol and water) stimulated the 2-NBDG uptake into adipocytes in a concentration-dependent manner. There was a higher uptake of 2-NBDG in the presence of methanol extract at concentrations of 5–10 µg/mL, but this was significantly higher with the concentration of 15 µg/mL compared to that of insulin. The 2-NBDG uptake in the presence of 15 µg/mL of methanol extract was 1.41-fold higher than insulin (*p* < 0.05). On the other hand, the uptake of 2-NBDG in the presence of water extract at the concentration of 5 µg/mL was 0.42-fold lower than insulin (*p* < 0.05).

Nevertheless, with concentrations of 10 and 15 µg/mL, the uptake in the presence of the water extract was almost the same as the insulin. In the same way, at the concentrations of 5 and 10 µg/mL, the 2-NBDG uptake into adipocyte cells in the presence of rosiglitazone was up to 3.67- and 1.44-fold higher, respectively, than the water (*p* < 0.05) extracts. However, no notable difference in the 2-NBDG uptake was observed between cells treated with rosiglitazone and those treated with 15 µg/mL of the methanol extract. Moreover, glucose uptake was increased significantly in the presence of 10 and 15 µg/mL of water extract and all concentrations of the methanol extract compared to the untreated cells. At the 5 µg/mL concentration, the glucose uptake was 0.78- and 1.79-fold higher for the water and methanol extracts, respectively, compared to the negative control. Nevertheless, the 2-NBDG uptake into adipocyte cells in the presence of methanol extract and the water extract was quite different; the methanol extract displayed 1.21-fold higher uptake than the water extract at the concentration of 15 µg/mL.

### 2.4. DPPH and FRAP Assay

The antioxidant assays (DPPH and FRAP) measured for the water and methanol extracts of *P. malayana* leaves are shown in Table 2. Methanol extract of *P. malayana* leaves exhibited potential antioxidant activity, evidenced by its low DPPH radical scavenging IC_50_ and high FRAP values. The DPPH radical scavenging activity and ferric reduction activity followed a similar pattern with the highest activity of the methanol extract. In both assays, the values of methanol and water extracts were significantly different (*p* < 0.05). Water extract showed the lowest scavenging action with the highest IC_50_ value of 27.12 μg/mL compared to methanol extracts (10.85 μg/mL) and ascorbic acid (5.08 μg/mL). Similarly, the lowest FRAP activity was observed in water extract with 33.71 mg of ascorbic acid equivalent (AAE) per gram of sample, indicating lower antioxidant activity than the methanol extract (72.53 mg AAE/g).

### 2.5. Toxicity Study

#### 2.5.1. Morphological Defects Related to Mortality of Zebrafish Embryos Exposed to the *P. malayana* Extracts

Different ratios of aqueous-methanol were used because methanol and water extract showed potential antidiabetic and antioxidant activities. The fine-tuning of the solvent ratios aimed to observe a broader view of the toxicity window. Coagulation, lack of somite formation, non-detachment of the tail, and lack of a heartbeat are the apical observations for determining lethality. Any positive detection of one of these observations leads to a lethal decision [27]. The summary of the apical observations in zebrafish exposed to all extracts along with the solvent control (0.1% dimethyl sulfoxide in E3 medium), positive control (4 mg/L of 3,4-dichloroaniline in E3 medium), and negative control (E3 medium) groups is shown in Table 3. It was found that the lowest concentrations of all extracts showed no apical observations. Similarly, the negative and solvent control showed no lethal defects. However, coagulation, non-detachment of the tail, and lack of a heart were observed in the embryos exposed in the positive control. The high concentration of all extracts exhibited embryo coagulation, but non-detachment of the tail and lack of a heartbeat were only detected in the embryo treated with the high concentration of the 100% methanol extract. In this study, the same concentration could not be used for all extracts because the highest concentration of every extract should have 100% mortality, whilst the lowest concentration should provide no observable effect [27].

**Figure 3:**
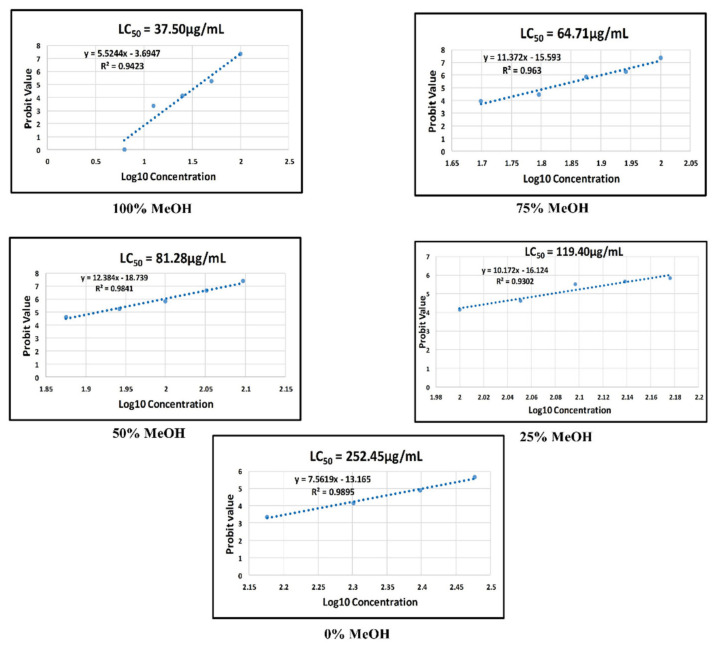
Logarithmic measurement of the LC_50_ value of five *P. malayana* leaf extracts (0, 25, 50, 75, and 100% *v/v* methanol-water) using probit analysis.

The logarithmic measurement of the median lethal concentration value for five extracts (0, 25, 50, 75, and 100% *v/v* methanol-water) is shown in Figure 3. The LC_50_ value for the 100% *v/v* methanol extract was 37.50 µg/mL (or 37.50 mg/L). The LC_50_ values of the water, 25%, 50%, and 75% *v/v* methanol extracts were calculated as 252.45, 119.40, 81.28, and 64.71 µg/mL, respectively. All teratogenic parameters were then analyzed for the one with the lowest LC_50_ value, the 100% *v/v* methanol extract.

#### 2.5.2. Mortality and Other Morphological Defects of Zebrafish Embryo Exposed to 100% Methanol Extract

##### Mortality

The mortality rate of six different concentrations of 100% *v/v* methanol extract was observed along with the solvent control (0.1% dimethyl sulfoxide in E3 medium), positive control (4 mg/L of 3,4-dichloroaniline in E3 medium), and negative control (E3 medium) as shown in Table 4. The positive control and the embryo treated with the plant extract at the concentration of 12.5 µg/mL and above exhibited the sign of mortality. On the other hand, the negative control, solvent control, and embryo treated with the plant extract at the concentration of 6.25 and 3.125 µg/mL showed a 100% survival rate. The other morphological defects unrelated to the lethal category were evaluated in the zebrafish with a 100% survival rate, as shown in Table 5.

##### Hatchability

Generally, healthy embryos start to hatch by 48 hpf and finish by 72 hpf, suggesting the embryo’s normal growth into larvae [27]. The hatching defect was observed throughout this analysis in the embryo treated with >6.25 µg/mL of the plant extract, similar to that of the positive control (Table 4 and Table 5). However, it was not observed in the embryo treated with 3.125 µg/mL plant extract and negative/solvent controls.

##### Yolk and Eye Size

**Figure 4:**
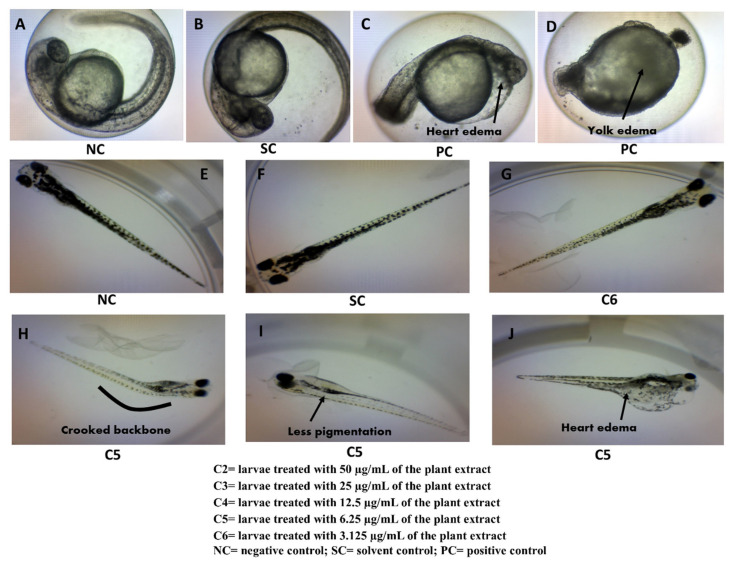
Morphological defect in zebrafish embryo/larvae. (**A**) NC/healthy embryo at 24 hpf; (**B**) embryo in SC at 24 hpf; (**C**) heart edema observed in the PC embryo; (**D**) yolk edema observed in the PC embryo; (**E**) NC/healthy larvae; (**F**) larvae in the solvent control; (**G**) larvae treated with C6; (**H**) crooked backbone observed in the larvae treated with C5; (**I**) less pigmentation observed in the larvae treated with C5; (**J**) heart edema observed in the larvae treated with C5.

The yolk and eye size were analyzed using DanioScopeTM software, and the results are shown in Table 5. The yolk size, measured at 24 hpf, showed no significant difference (*p* < 0.05) at all extract concentrations compared to those of the negative and solvent controls. In contrast, the positive control exhibited yolk edema, as shown in Figure 4D. However, the eye size of the embryo treated with 6.25 µg/mL of the plant extract was significantly bigger (*p* < 0.05) than those of the negative and solvent controls, while the eye size of the positive control was significantly reduced (*p* < 0.05) compared to the controls.

##### Pigmentation Defect, Crooked Backbone, and Heart Edema

**Figure 5:**
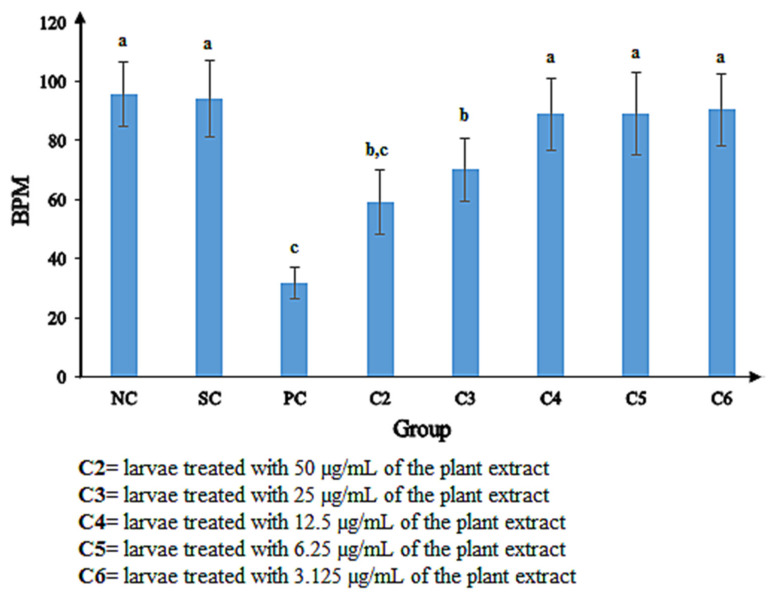
Heartbeat per minute (BPM) of zebrafish embryo/larvae exposed to different concentrations of methanol extracts along with the negative, solvent, and positive control. C2–C6 = treatment concentrations, NC = Negative control; SC = Solvent control; PC = Positive control. Different letters (a,b,c) indicate significant differences (*p* < 0.05) among samples.

The pigmentation defect (Figure 4I, Table 5), crooked backbone (Figure 4H, Table 5), and heart edema (Figure 4J, Table 5) were detected in the larvae treated with 6.25 µg/mL of the plant extract. The positive control exhibited heart edema (Figure 4C) and yolk edema (Figure 4D). Due to the hatching defect, the pigmentation defect and crooked backbone could not be analyzed in the positive control. All the aforementioned defects were not present in the negative and solvent controls. The heart rate (BPM) of different concentrations of 100% methanol extract was observed along with the solvent control (0.1% dimethyl sulfoxide in E3 medium), positive control (4 mg/L of 3,4-dichloroaniline in E3 medium), and negative control (E3 medium) as shown in Figure 5. There was no notable difference observed in the heart rate among C4 (12.5 μg/mL), C5 (6.25 μg/mL), and C6 (3.125 μg/mL) compared to the negative control, whereas BPM was significantly reduced in the embryos exposed to the higher concentration of methanol extracts (25 and 50 μg/mL) than in the negative control (NC). In addition, there was no notable difference in the heart rate observed between the groups of embryos exposed to C2 (50 μg/mL) and the positive control (PC). Due to early mortality, the heart rate could not be observed in the embryos exposed to the highest concentration of methanol extract (100 μg/mL).

##### Body Length

The body length of the larvae measured at the stage of 96 hpf is shown in Table 5. In this study, the average body length for the negative control group was 2.8 mm. The body length of the embryos exposed to 6.25 and 3.125 µg/mL was significantly lower than the negative and solvent controls. However, the body length of the positive control could not be analyzed due to the hatching defect.

### 2.6. GC-MS Analysis after Derivatization

**Figure 6:**
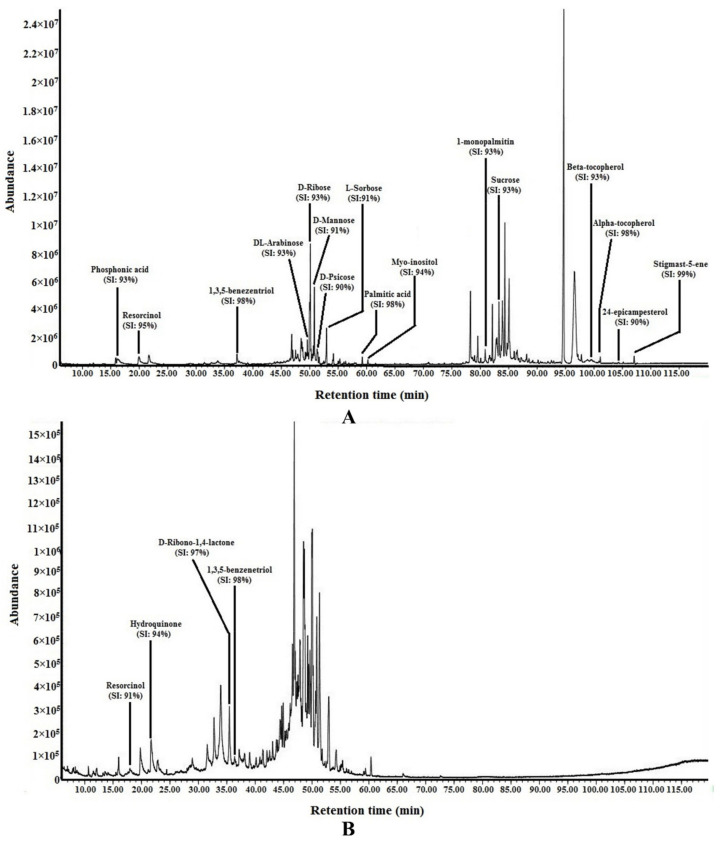
GC-MS chromatogram of derivatized methanol extract (**A**) and water extract (**B**) of *P. malayana* leaves.

The putative compounds detected in the derivatized methanol and water extracts of *P. malayana* leaves were confirmed by comparing the fragment *m*/*z* spectrum of each compound to the mass spectra database of the National Institute of Standards and Technology (NIST) 14 (Figures S1–S18). The GC-MS analysis after derivatization provided partial information on the chemical composition of the extracts. The name of the putative compounds, molecular formula, retention time, % of peak area, and similarity index (SI) are shown in Table 6. Various phytoconstituents with different categories were found, such as inorganic acid (phosphonic acid), simple sugars (DL-arabinose, D-ribose, D-psicose, sucrose, D-mannose, L-sorbose), sugar derivative (D-ribino-1,4-lactone), free fatty acid (palmitic acid), sugar-alcohol (myo-inositol), phenolics (beta-tocopherol, alpha-tocopherol, resorcinol, 1,3,5-benzenetriol, hydroquinone), glyceride (1-monopalmitin), and terpene (stigmast-5-ene). The SI of all these compounds was ≥90%. All the putative compounds were marked at the respective peaks in the representative GC-MS chromatogram of the methanol and water extracts of *P. malayana* leaves (Figure 6).

## 3. Discussion

In this study, the trend for extraction yield was methanol > water > ethyl acetate > *n*-hexane, indicating that the major phytochemicals present in *P. malayana* leaves are polar and soluble in methanol. On the other hand, the lowest yield of hexane extract indicated that the major phytochemicals present in *P. malayana* leaves are polar.

The methanol extract showed the maximum AGI activity (IC_50_ 2.71 µg/mL) among other extracts. This finding is not in line with Jemain et al. [28], who reported that no alpha-glucosidase inhibition had been shown by *P. malayana* leaf. A possible explanation for this discrepancy is that they utilized maceration as an extraction technique and dichloromethane as an extraction solvent. As established in a previous study, different absorbance conditions, extraction procedures, and solvents can affect the extraction yield and plant bioactivity [29,30,31,32].

3T3-L1 cell lines have been used for glucose uptake activity evaluation since after differentiation to adipocytes, 3T3-L1 preadipocytes are effective for investigation of glucose metabolism [33,34]. The insulin-treated cell showed a higher amount of glucose uptake compared to the untreated cell. Litwack [35] reported that insulin could stimulate the glucose uptake by GLUT4 in the cells and ultimately reduce the blood glucose level. On the other hand, rosiglitazone exhibited higher uptake than both the negative control and insulin-treated 3T3-L1 cells. It could be due to the insulin-sensitizing activity of rosiglitazone [36]. In the presence of methanol extract, the 2-NBDG glucose uptake into the 3T3-L1 adipocyte cell was higher than water extracts, suggesting the strong antidiabetic potential for methanol extract compared to water extract. These results align with the present findings in which methanol extract showed potent AGI and antioxidant activities. Therefore, 15 µg/mL of methanol extract was as potent as the rosiglitazone in sensitizing the 3T3-L1 cells to insulin by making the cells more responsive. Although the water extract does sensitize the 3T3-L1 cells, its potency was significantly lower than rosiglitazone, even with a high concentration at 15 µg/mL. Therefore, it can be summarized that the *P. malayana* leaf methanol extract exhibited insulin-sensitizing activity; thereby, it could be considered as a potential antidiabetic agent.

From the DPPH and FRAP findings, it was observed that both the AGI and antioxidant assay followed a similar trend for methanol and water extracts. However, methanol extract exhibited the highest activity compared to water extract. The organic solvent has been reported to show a better extractive capability compared to water for the extraction of some bioactive compounds [37]. This result was in line with the previous findings, where the methanol extract displayed high antioxidant activity in contrast to the water extract [38]. Both assays were performed to cover a wide range of compounds. DPPH assay only measures hydrophobic antioxidants, whereas FRAP for that of hydrophilic [39].

Palmitic acid, 1,3,5-benzenetriol, 1-monopalmitin, beta-tocopherol, 24-epicampesterol, alpha-tocopherol, and stigmast-5-ene were detected as alpha-glucosidase inhibitors in our previous research [6]. On the other hand, palmitic acid, isolated from the methanolic fraction of *Syzygium littorale* stem bark, was reported as an antioxidant (IC_50_ 189.9 μg/mL) [40]. Additionally, tocopherols are natural antioxidants that can prevent lipid oxidation by scavenging free radicals by interacting with singlet oxygen. Beta-tocopherol shows higher antioxidant capacity compared to alpha-tocopherol at high temperatures [41]. Furthermore, hydroquinone is a phenolic compound commonly used in cosmetics as an antioxidant [42], found in the water extract of *P. malayana* leaves. Besides this, resorcinol, found in both methanol and water extracts of *P. malayana*, is used as an antioxidant to prevent the browning of frozen, fresh, and deep-frozen crustaceans (crab, shrimp, lobster) [43]. Moreover, Yoshida and Niki [44] reported the antioxidant activity of epicampesterol. Based on these previous studies, the presence of tocopherols, palmitic acid, hydroquinone, resorcinol, and 24-epicampesterol might be the possible reason for the potent antioxidant activity of *P. malayana* methanol and water extracts. Besides antioxidant activity, alpha-tocopherol (vitamin E) enhanced insulin sensitivity in overweight diabetic patients [45]. On the other hand, seven alkaloids (hodgkinsine, calycanthine, (+/−)-chimonanthine, meso-chimonanthine, 2-ethyl-6-methylpyrazine, and 3-methyl-1,2,3,4-tetrahydro-γ-carboline) from the methanol extract of *P. malayana* leaves as reported by Hadi et al. [3] have not been investigated for their AGI, antioxidant, and insulin-sensitizing activities. Further study is required to investigate the phytoconstituents responsible for the insulin-sensitizing effect of *P. malayana* leaf extracts.

The 100% methanol extract exhibited the lowest LC_50_ value compared to other extracts (LC_50_ = 37.50 µg/mL). It was observed that the LC_50_ value decreased when the percentage of methanol used as a solvent increased during extraction. However, the LC_50_ value was affected by the metabolites in the extract and no interference by the methanol. GC-MS did not detect the remaining methanol in the extract. Furthermore, the extract was adequately dried through a rotary evaporator and freeze dryer.

The methanol extract of *P. malayana* was earlier reported to contain alkaloids, such as hodgkinsine, calycanthine, (+/−)-chimonanthine, meso-chimonanthine, 2-ethyl-6-methylpyrazine, and 3-methyl-1,2,3,4-tetrahydro-γ-carboline [3]. These compounds have previously been reported to exert various bioactivities without demonstrating any toxicity [46,47,48]. Although various bioactivities were reported on these alkaloids, no report on their antidiabetic activity has been found. Unfortunately, the GC-MS failed to detect these compounds in the current study, which may be due to the non-volatile nature of these compounds. However, 10 different types of putative compounds were detected in *P. malayana* methanol extract through GC-MS analysis after derivatization in the current study. Partial information on the chemical composition of the extracts was obtained. Among these compounds, palmitic acid, phosphonic acid, 1,3,5-benzenetriol, stigmast-5-ene, and alpha-tocopherol were detected as the major compounds. The previous study reported that palmitic acid, stigmast-5-ene, and 1-monopalmitin might contribute to the toxic effect, which is in line with the findings of this present study [18]. Some studies detected the harmful effects of palmitic acid. Palmitic acid may be considered to be playing as a high-risk factor for coronary heart disease [49]. *P. malayana* methanol extracts at a concentration of 50 and 25 µg/mL showed a lower heartbeat than the negative control, which might be attributed to the presence of palmitic acid since it was detected as one of the major compounds in the extract. Palmitic acid has been reported to show a toxic effect in B16F10 murine melanoma cell lines by causing both apoptosis and necrosis. Furthermore, it also showed a cytotoxic effect in SK-Mel 23 cell lines at a concentration of 200 µm by causing DNA fragmentation [50]. Another study was also found to cause hepatic steatosis at 0.25 and 0.5 mmoL/L and exerted a dose-dependent cytotoxic effect by inducing apoptosis and necrosis, and decreasing albumin production [51].

Phosphonic acid and alpha-tocopherol were also found in abundance in the *P. malayana* leave methanol extract. Phosphonic acid is also known as phosphorous acid, marketed widely as a fertilizer and pesticide [52]. Though alpha-tocopherol (vitamin E) is an essential nutrient, the higher dose of alpha-tocopherol has been reported to cause epithelial dysfunction and increased intimal proliferation in male New Zealand White rabbits [53]. Intimal proliferation is a significant cause of restenosis (narrowing of the blood vessel) [54]. Due to the narrowing of a blood vessel, the blood circulation system may disrupt the overall growth of zebrafish, which clearly explains the significant decrease of the eye size and body length of the embryos at higher concentrations compared to normal. The toxicity effects of the remaining compounds found in the *P. malayana* extracts have not been investigated; thus, it is recommended to be evaluated in future studies.

The 100% methanol extract exerted the lowest LC_50_ values compared to other extracts; hence, all teratogenic parameters were analyzed for this extract. The yolk size, eye size, and body length of the embryos at different extract concentrations are shown in Table 5. The yolk size of the embryos was not affected after treatment with the extract except the positive control. On the other hand, the eye size was affected by the plant extract and the positive control while the body length was significantly decreased at both concentrations of *P. malayana* extracts compared to the normal. Body length has been reported to be inhibited due to the inhibition of thyroid peroxidase hormone or thyroid peroxidase-like activity in zebrafish embryos [55].

The hatchability of zebrafish embryos showed a dose-dependent reduction when exposed to *P. malayana* extracts (Table 4), which indicated the developmental toxicity. The hatchability of the embryos might be retarded due to two reasons. One is the disturbance of the hatching enzyme by which chorion is digested, and another reason is hypoxia since oxygen is essential for embryonic development [56].

During embryonic development phases, the heartbeat of embryos is increased to maintain the blood flow of all organs. The present study revealed that the extract exhibited cardiovascular toxicity in zebrafish embryos. The heartbeat of the embryos was affected and found to be a concentration dependent (Figure 5), which is in line with previous studies [18,57,58,59]. The present study also detected the embryo’s heart edema and eye size defect, receiving 6.25 µg/mL of the extract. Mccollum et al. [60] reported that the failure of lipid metabolism and obstruction of the water permeability barrier of the *Danio rerio* embryo surface causes both defects.

Skeletal development is another crucial parameter for embryonic toxicity assessment. A crooked backbone was observed in the embryos exposed to 6.25 µg/mL (Table 5). Generally, skeletal development occurs after 72 hpf. At 24 hpf, the vertebrae column cells of the embryo migrate from the ventral somite and differentiate into the cartilaginous lineage, and the first cranial cartilage is developed at 24 hpf. Since skeletal cells are developed from somite, the crooked backbone might appear due to early defects in somite formation [60,61].

It becomes crucial to calculate the dose during a prescription to avoid side effects, considering that the therapeutic dose should be lower than the LC_50_ value. The present study revealed that the IC_50_ values of the methanol extract on AGI activity and DPPH were 5.37 and 10.85 μg/mL, respectively, while the IC_50_ values of the water extract on both activities were 6.75 and 27.12 μg/mL. In addition, there was a significantly higher uptake of 2-NBDG by the treated 3T3-L1 cells in the presence of methanol and water extract at concentrations of 5 and 10 µg/mL, respectively, compared to that of insulin. These therapeutic values of methanol and water extracts were much lower than the LC_50_ values: 37.5 and 252.5 μg/mL, respectively. It indicates that both extracts are safe to use and are potentially antidiabetic agents. Nevertheless, the LC_50_ value of the water extract was almost 9 times higher than the methanol extract, and its therapeutic values were comparable to that of methanol. In addition, water is more cost-effective than methanol to be used as a solvent during extraction. It makes the water extract preferable over the methanol extract as a potential antidiabetic agent.

## 4. Materials and Methods

### 4.1. Chemicals and Reagent

The organic chemicals of analytical grade, namely ethanol, methanol, *n*-hexane, ethyl acetate, and dimethyl sulfoxide (DMSO), were obtained from Merck (Darmstadt, Germany). The four inorganic salts (sodium chloride, potassium chloride, calcium chloride, and magnesium sulfate) and methylene blue were purchased from Merck (Darmstadt, Germany). Ascorbic acid, rosiglitazone, ρ-nitrophenyl-ρ-D-glucopyranosidase (PNPG), quercetin, insulin, pyridine anhydrous, methoxamine hydrochloride, *N*-methyl-*N*-(trimethylsilyl) tryfluoroacetamide (MSTFA), and phosphate-buffered saline (PBS) were bought from Sigma-Aldrich (St. Louis, MO, USA). The AG enzyme (source: yeast maltase) was purchased from Megazyme, Ireland. Dulbecco’s modified eagle medium (DMEM) and fetal bovine serum (FBS) were purchased from Gibco, Life Technologies (Carlsbad, CA, USA). Furthermore, the 2-NBDG Glucose Uptake assay kit and tetrazolium salt (3-(4,5-dimethylthiazol-2-yl)-2,5-diphenyltetrazolium bromide (MTT) were procured from Abcam (Cambridge, UK) and Life Technologies (Carlsbad, CA, USA), respectively. The 3T3-L1 cell line was obtained from American Type Culture Collection ATCC (Manassas, VA, USA).

### 4.2. Collection of Sample and Plant Extract Preparation

Fresh leaves of *P. malayana* were collected from Jambi, Indonesia, and authenticated by a taxonomist, Dr. Shamsul Khamis, Department of Botany, University Putra Malaysia, Malaysia. The plant sample was deposited in the Herbarium, Kulliyyah of Pharmacy, IIUM (voucher specimen #PIIUM008-2). The leaves were initially cleaned and allowed to dry under a shade at room temperature (25 ± 5 °C) for seven days and then pulverized by utilizing a universal cutting mill (Fritsch, Germany) to obtain powdered leaves and stored at −80 °C before the extraction process [37].

Leaf powder (1 g) was subjected to extraction with sonication for 30 min using a sample-to-solvent ratio of 1:3 (*w*/*v*). For preliminary screening of the AGI activity, four different types of solvents based on the different degrees of polarity index, namely *n*-hexane, ethyl acetate, methanol, and water, were initially used as extraction solvents. Extraction was done with the combination of water and methanol using various ratios (0, 25, 50, 75, and 100% *v*/*v*). The Whatman grade No. 1 filter paper was used for filtration purposes. The remaining solvent was allowed to evaporate using a rotary evaporator at 40 °C. Subsequently, the crude extract was freeze-dried to eliminate the residual moisture and preserved at −80 °C [37]. The following equation was used to calculate the % of yield:

Yield of extraction (%, *w*/*w*) = Wt_1_/Wt_2_ × 100% (1)

where Wt_1_ and Wt_2_ represent the final weight of the dried extract and the primary weight of the leaf powder [62].

### 4.3. AGI Activity

The AGI activity was investigated by following the method described by Murugesu et al. [62] with slight modifications. First, 2 mg of quercetin, the positive control, were dissolved in 1 mL of DMSO, whereas the substrate, PNPG, was prepared by dissolving 6 mg in 20 mL of 50 mM phosphate buffer and adjusted pH at 6.5. The yeast, glucosidase, was used in this study under the consideration that its substrate specificity, inhibitor sensitivity, and optimum pH are similar to the mammalian glucosidase [63]. The sample was prepared following the same preparation protocol for quercetin. While the negative control was prepared by replacing the sample with DMSO. A total of 10 µL of the samples, quercetin, and DMSO; 100 µL of 30 mM phosphate buffer; and 15 µL of 0.02 U/µL AG enzyme were transferred into the 96-well plate. A blank was prepared following the same protocol but without the enzyme. The samples and the blank mixture were both incubated at room temperature for 5 min and then treated with 75 μL of PNPG, followed by addition of glycine (pH 10) and another incubation time of 15 min at room temperature to stop the reaction. The microplate reader (Tecan Nanoquant Infinite M200, Tecan, Männedorf, Switzerland) was used to record the absorbance reading at 405 nm. The linear regression analysis was used to obtain the IC_50_. The IC_50_ value is a useful measure for determining an inhibitor’s efficacy and potency; it shows the quantity of inhibitor required to halve the reaction [64]. All tests were carried out in triplicate. The following equation was used to calculate the AGI activity:

AGI activity (%) = [(A_control_ − A_sample_)/A_control_] × 100 (2)

where A_sample_ and A_control_ represent the sample’s or positive control’s and negative control’s absorbance, respectively [65].

### 4.4. 2-NBDG Uptake in 3T3-L1 Cells Assay

#### 4.4.1. Cell Culture

The 3T3-L1 cells (ATCC, Manassas, VA, USA) were cultured in complete growth DMEM media containing 10% FBS and 1% penicillin-streptomycin. The 3T3-L1 cells were kept at 37 ± 0.5 °C in a humidified environment containing 5% CO_2_. It was sub-cultured every 3 to 4 days at approximately 80 to 90% confluence [66].

#### 4.4.2. Cell Viability Assay

The methanol and water extracts of *P. malayana* leaves were tested for their cytotoxicity effect by determining the metabolic activity of 3T3-L1-treated cells compared to the negative control (untreated 3T3-L1 cells) via the MTT (3-(4,5-Dimethylthiazole-2-Yl)-2,5-Diphenyltetrazolium Bromide) assay according to the method reported by Al-Zikri et al. [67] with some modifications. A total of 1.0–2.0 × 10^5^ cells/mL were seeded in a 96-well plate overnight at 37 °C with a 5% CO_2_ atmosphere. Then, the 3T3-L1 cells were treated with serial dilution concentrations of the extracts (3.125, 6.25, 12.5, 25, 50, and 100 µg/mL) in triplicate for 24 h at 37 °C with a 5% CO_2_ atmosphere. Solvent (vehicle) used to dissolve the sample was used at a final concentration of <0.1%, which had no impact on the assay. After 24 h, all media were discarded, and in each well, 30 μL of MTT solution (5 mg/mL MTT in phosphate-buffered saline) were added. The formazan crystals were dissolved in 100 μL of DMSO and left in the dark at room temperature for a further 1 h after being incubated with MTT solution for 4 h. A microplate reader (Tecan Nanoquant Infinite M200, Männedorf, Switzerland) was used to detect absorbance at 570 and 630 nm, which served as the reference wavelength. Untreated 3T3-L1 cells served as the negative control, while wells containing media along with 0.1% (*v*/*v*) DMSO served as the solvent control. After blank subtraction, the results were represented as a percentage of the average absorbance of extract-treated 3T3-L1 cells compared to untreated 3T3-L1 cells. IC_50_ was determined based on the linear regression (y = mx + c) analysis using excel, where the straight line was developed by plotting the concentration (*X*-axis) versus percentage of cell viability (*Y*-axis). The following equation was used to calculate the percentage of cell viability:

Cell viability (%) = [(A_Sample_ − A_Blank_)/(A_Untreated_ − A_Blank_)] × 100 (3)

where A indicates the absorbance.

#### 4.4.3. 2-NBDG Uptake in 3T3-L1 Adipocyte Cells

The 2-NBDG uptake assay was performed by following the procedure published by Hasan et al. [68] with minor modifications. In brief, preadipocytes were treated with an adipogenic cocktail to induce differentiation into mature adipocytes. The 3T3-L1 cells were seeded at a density of 1.0–2.0 × 10^5^ cells/mL into a 96-well plate and maintained the incubation temperature at 37 °C with 5% CO_2_ atmosphere until it attained confluence. On day 2 post-confluence (Day 0), the 3T3-L1 cells were stimulated to differentiate using differentiation cocktail DMEM (MDI) supplemented with 10% FBS, 1 µg/mL of insulin, 0.25 µM of dexamethasone, and 0.5 mM IBMX (phosphodiesterase inhibitor methylisobutylxanthine). On day 2 of differentiation, the differentiation medium was replaced with maintenance media, which contained 1 µg/mL of insulin in complete DMEM, and further incubated for another 2 days (day 4). On day 5, the medium was replaced with complete DMEM, and the same medium was used until day 8.

Post cell differentiation (day 8), the 3T3-L1 cells were starved for two days by incubating them in serum- and glucose-free DMEM. Afterward, the serum and glucose starving adipocytes were treated with a final concentration of 100 µg/mL of 2-NBDG, as is mentioned in Table 7, in glucose-free media for 24 h. After 24 h, the cultures were washed with PBS to get rid of 2-NBDG, and the fluorescence of the cell monolayer was measured at the excitation wavelength (485 nm) and emission wavelength (535 nm) using a microplate reader.

### 4.5. In Vitro Analysis of Antioxidant Activity

#### 4.5.1. DPPH Assay

The DPPH scavenging assay was conducted following the method mentioned by Khatib et al. [69] with minor modifications. Seven concentrations (200, 100, 50, 25, 12.5, 6.25, and 3.125 μg/mL) of samples were prepared by serial dilution for this test. Sample solutions were prepared by dissolving in methanol. DPPH solution (0.2 mM) was also prepared with methanol. In a 96 well-plate, 20 μL of sample solution were added to 80 μL of DPPH solution and subjected to incubation for around 10 min in a dark place. Here, ascorbic acid served as a positive control, and the blank was absent from the DPPH solution. The absorbance was recorded at 540 nm using a microplate reader (Tecan Nanoquant Infinite M200, Tecan, Männedorf, Switzerland). The following equation was used to calculate the DPPH scavenging activity (%):

Percentage of DPPH scavenging activity = (A_control_ − A_sample_)/A_control_ × 100 (4)

where A_sample_ and A_control_ represent the sample’s or positive control’s and negative control’s absorbance, respectively [65].

#### 4.5.2. FRAP Assay

The protocol described by Khatib et al. [69] was carried out with some modifications. The freshly prepared FRAP reagent was a mixture of 2.5 mL of 10 mM 2,4,6-tris(2-pyridyl)-S-pyrazine in 40 mM hydrochloric acid, 2.5 mL of 20 mM ferric chloride, and 25 mL of 0.1 M acetate buffer with pH 3.6. Then, this reagent was incubated at 37 °C for 10 min. In a 96-well plate, a mixture of 20 μL of sample solution (prepared in methanol), 40 μL of freshly prepared FRAP reagent and 140 μL of distilled water was prepared, which produced a blue color. The mixture was incubated for 20 min in a dark place, and the absorbance was recorded at 593 nm using a microplate reader (Tecan Nanoquant Infinite M200, Tecan, Männedorf, Switzerland). Ascorbic acid was used as a standard to develop the standard calibration curve. The blank was prepared with 40 μL of FRAP reagent in 200 μL of distilled water. The FRAP activity was calculated by interpolation of the net absorbance of the calibration curve and stated as the ascorbic acid equivalent milligram of ascorbic acid per gram of sample (mg AAE/g).

### 4.6. Toxicity Test

In this study, the OECD guidelines were duly followed to observe the toxic effects of *P. malayana* leaf extracts. The breeding procedures and maintenance of *Danio rerio* were also carried out following the same guidelines [27].

#### 4.6.1. Maintenance of Zebrafish

Wild-type adult zebrafish were purchased and maintained at the Central Research and Animal Facility (CREAM) IIUM, Kuantan, Malaysia, and the registered ethical approval number, IIUM/IACUC Approval/2016/(12) (85). All the fish were free of macroscopically noticeable symptoms of infection and disease and did not use for any pharmaceutical (acute or prophylactic) treatment for two months before spawning and were maintained in aquaria with a loading capacity of 1 L of water per fish and a fixed 12–16 h photoperiod [25]. The filtration system and water quality were allowed to meet all the specifications recommended by OECD guidelines. The water temperature was maintained at 26 ± 1 °C. Dry flake food and artemia were given three times daily, and excess food and feces were removed one hour after feeding to ensure optimal water quality. The fish holding tanks were housed in a closed system of a multi-rack aquarium. The multi-rack aquarium system was maintained at the Zebrafish Laboratory in CREAM, IIUM.

#### 4.6.2. The Breeding Procedure of *Danio rerio* and Maintenance of Embryo

The experiment was done using synchronized aged zebrafish eggs from a controlled breeding method. The male and female (2:1) matured fish were separated and only allowed to mate when the light was first turned on [27]. Upon fertilization, eggs were taken from the breeding tanks, combined, and picked randomly for testing [27]. The eggs were washed three times to remove debris with E3 medium prepared by dissolving four types of salts (0.292 g of 5.0 mM NaCl, 0.013 g of 0.17 mM KCl, 0.044 g of 0.33 mM CaCl, and 0.081 g of 0.33 mM MgSO_4_) in 1 L of distilled water and methylene blue (100 μL) was added to inhibit the growth of fungus [70]. The damaged and dead embryos were withdrawn using a pipette with a widened opening. The eggs then shifted to a clean E3-containing petri dish and were kept in the incubator at 26 ± 1 °C [27]. For the observation of embryonic growth, the microscopic examination was conducted at 6 h post-fertilization (hpf) before sample treatment.

#### 4.6.3. Preparation of the Sample and Experimental Procedure

The healthy fertilized embryos were moved to a 96-well plate at 6 hpf by using a sterile pipette attached with a 1 mL micropipette tip. Each well was occupied by one embryo. The experiment was conducted with 20 eggs for each test concentration, 20 eggs for the positive control (4 mg/L of 3,4-dichloroaniline in E3 medium), 20 eggs for the solvent control (0.1% dimethyl sulfoxide in E3 medium), 20 eggs for the negative control (E3 medium), and 4 eggs as the internal plate control for each plate. The total volume per well was 300 µL for each experimental group, consisting of 150 µL of E3 medium aspirated with an embryo at 6 hpf and 150 µL of the sample prepared in 0.1% DMSO. For 0% *v/v* methanol extract of *P. malayana* leaves, the final concentrations were 50, 100, 150, 200, 250, 300, and 350 µg/mL in E3 medium. For 25% *v/v* methanol extract, the final concentrations were 62.5, 75, 87.5, 100, 112.5, 125, 137.5, 150, 162.5, and 175 µg/mL in 0.1% DMSO. For 50% *v/v* methanol extract, the yielded final concentrations were 12.5, 25, 37.5, 50, 62.5, 75, 87.5, 100, 112.5, and 125 µg/mL in 0.1% *v/v* DMSO. For 75% *v/v* methanol extract, the final concentrations were 12.5, 25, 37.5, 50, 62.5, 75, 87.5, and 100 µg/mL in 0.1% *v/v* DMSO. For 100% *v/v* methanol extract, the final concentrations were 3.125, 6.25, 12.5, 25, 50, and 100 µg/mL in 0.1% *v/v* DMSO. All plates were incubated at a temperature of 26 ± 1 °C [27].

#### 4.6.4. Microscopic Observations

The percentage of mortality and LC_50_ were observed and calculated for all extracts. According to the OECD guidelines, four parameters indicated lethality, such as coagulation, lack of somite formation, non-detachment of the tail, and lack of a heartbeat.

Probit analysis was used to determine the LC_50_ [18,58,71]. It is the statistical measurement of the concentration of toxicant (μg/mL or mg/L) needed to cause the death of 50% of the animals tested within the test duration [27]. The rates of mortality were calculated using the following equation:

Rate of mortality (%) = Number of dead embryos/Initial no. of embryos × 100(5)

The extract with the lowest LC_50_ was examined further to detect the other abnormalities of the hatching rate, yolk size, eye size, crooked backbone, pigmentation, heart edema, yolk edema, and body length. A Nikon light microscope (Nikon Corporation, Tokyo, Japan) was used to observe the embryos. The survival and sublethal endpoint were assessed during the treatment period (24, 48, 72, and 96 hpf). The heartbeat was video recorded and pictured using a Dino-eye eyepiece camera and Nikon light microscope, respectively. Both videos and images were analyzed using non-invasive DanioScope software (Noldus Information Technology, Wageningen, The Netherlands).

### 4.7. Identification of Compounds from Methanol Extracts of P. malayana Leaves by GC-MS Analysis after Derivatization

The extracts of *P. malayana* were derivatized, and the putative compounds were identified following the procedure mentioned by Javadi et al. [37] with slight modifications. For derivatization, approximately 25 mg of plant extract were dissolved in 50 μL of pyridine. Prior to incubation for 2 h at 60 °C, 100 μL of methoxamine hydrochloride solution were added into the mixture, followed by the addition of 300 μL of MSTFA and allowed to incubate for another half an hour at 60 °C. Finally, the mixture was kept at room temperature (27 ± 1 °C) for overnight incubation. GC-MS (Agilent 6890) was connected to a selective mass detector (HP 5973) and equipped with a capillary column DB-5ms with a thickness, diameter, and length of 0.25 µm, 250 µm, and 30.0 m, respectively. The derivatized plant extracts were injected at 2 µL into the system in split-less mode. The initial oven temperature was set at 85 °C, which was increased to the target temperature of 315 °C at a rate of 2 °C per minute and held for 5 min. The flow rate for helium (carrier gas) was set at 0.8 mL/min. The mass scan parameters ranged from 50 to 550 *m*/*z*, and the fragment *m*/*z* spectra of each compound were compared with the National Institute of Standards and Technology (NIST) 14 database.

### 4.8. Statistical Analysis

Minitab 16 (Minitab Inc., State College, PA, USA) was used to perform the data analysis, and all data are represented as mean ± standard deviation (SD). For the yield of extraction, AGI, 2-NBDG glucose uptake in 3T3-L1 cell lines, and antioxidant assays (DPPH and FRAP), the number of replication (*n*) was 3. However, for the toxicity study, *n* = 20, according to the OECD guideline. The notable differences among the groups were calculated using one-way analysis of variance (ANOVA) together with a post hoc test, Tukey’s comparison test, where the significant level and confidence interval were set at *p* < 0.05 and of 95%, respectively. Data with different letters are significantly different. Data normality was checked using the Shapiro–Wilk test.

## 5. Conclusions

This study evaluated the AGI, insulin-sensitizing, and antioxidant activities, as well as the toxicity of *P. malayana* leaf extracts. The methanol and water extracts exhibited high inhibitory activity against the alpha-glucosidase enzyme. Both extracts also effectively improved the glucose uptake. Furthermore, both extracts exhibited potent antioxidant activities. The findings on the toxicity evaluation using zebrafish embryos revealed that the LC_50_ value of both extracts was much higher than their therapeutic concentration, indicating that both extracts are safe for use. The therapeutic index of water extract was higher than that of methanol since the LC_50_ values of the water extract were much higher than the methanol extract, while its therapeutic concentrations were almost similar to the methanol, although slightly higher. It makes water preferable for use as an extraction solvent; besides, water is more cost-effective than methanol. This finding provides a solid base to perform in vivo studies in the future. In addition, some putative compounds were identified by GC-MS analysis after derivatization to provide a scientific explanation and strengthen the findings. However, this finding is based on partial information on the chemical composition of the plant extract. Therefore, further investigation is required to isolate, identify, and quantify the bioactive compounds in relation to their antidiabetic activity and toxicity. The fractionation of the extract and the isolation of the bioactive compounds can be carried out through bioassay-guided fractionation and purification using different types of chromatographic techniques, such as liquid-liquid partition and solid-liquid chromatography (vacuum liquid chromatography, open column chromatography, and preparative high-performance liquid chromatography). The identification and quantification of compounds can be confirmed by multiple analytical instruments, such as nuclear magnetic resonance spectroscopy and liquid chromatography with tandem mass spectrometry (LC-MS/MS).

## Figures and Tables

**Table 1:** The AGI activity and yield of extraction of different extracts of *P. malayana* leaves.

Sample	AGI Activity IC_50_ (μg/mL)	% Yield (*w*/*w*)
Hexane extract	ND	1.06 ± 0.13 ^c^
Ethyl acetate extract	5.37 ± 0.14 ^b^	18.87 ± 0.93 ^b^
Methanol extract	2.71 ± 0.11 ^c^	31.51 ± 1.24 ^a^
Water extract	6.75 ± 0.16 ^a^	20.40 ± 1.47 ^b^
Quercetin	1.88 ± 0.003 ^d^	ND

Data with a different letter are significantly different with a *p*-value less than 0.05. Tukey’s multiple comparison test was used to calculate the data with a 95% confidence level. All the findings are displayed as mean ± standard deviation (*n* = 3). ND = not determined.

**Table 2:** DPPH and FRAP of methanol and water extracts of *P. malayana* leaves.

Sample	DPPH IC_50_ (μg/mL)	FRAP (mg AAE/g)
Methanol extract	10.85 ± 0.34 ^b^	72.53 ± 1.33 ^a^
Water extract	27.12 ± 0.95 ^a^	33.71 ± 1.84 ^b^
Ascorbic acid	5.08 ± 0.02 ^c^	ND

Data with a different letter are significantly different with a *p*-value less than 0.05. Tukey’s multiple comparison test was used to calculate the data with a 95% confidence level. All the findings are displayed as mean ± standard deviation (*n* = 3). ND = not determined.

**Table 3:** Morphological defects related to the mortality of zebrafish embryos exposed to different extracts of *P. malayana* at 24 to 96 hpf along with the solvent control (0.1% dimethyl sulfoxide in E3 medium), positive control (4 mg/L of 3,4-dichloroaniline in E3 medium), and negative control (E3 medium) groups.

Methanol-Water Extract	Extracts Concentration (µg/mL)	Coagulation	Non-Detachment of the Tail	Lack of Somite Formation	Lack of Heartbeat
100%	100, 50, 25, 12.5	+	+	−	+
	6.25, 3.125	−	−	−	−
75%	100, 87.5, 75, 62.5, 50, 37.5, 25	+	−	−	−
	12.5	−	−	−	−
50%	125, 112.5, 100, 87.5, 75, 62.5, 50, 37.5	+	−	−	−
	25, 12.5	−	−	−	−
25%	175, 162.5, 150, 137.5, 125, 112.5, 100, 87.5	+	−	−	−
	75, 62.5	−	−	−	−
0%	350, 300, 250, 200, 150	+	−	−	−
	100, 50	−	−	−	−
Controls	Negative control	−	−	−	−
	Solvent control	−	−	−	−
	Positive control	+	+	−	+

(+): present; (−): not present.

**Table 4:** Mortality rate (24 to 96 hpf) and hatching rate (72 hpf) of the developing zebrafish embryos treated with different concentrations of 100% *v/v* methanol extract of *P. malayana* leaves.

Plant Extract Concentration (µg/mL)	Mortality Rate (%) at 24 to 96 hpf	Hatching Rate (%) at 72 hpf
100	100	0
50	60	0
25	20	0
12.5	5	10
6.25	0	70
3.125	0	100
NC	0	100
SC	0	100
PC	90	5

NC: negative control; SC: solvent control; PC: positive control.

**Table 5:** Observations of eye size, yolk size, heartbeat, body length, hatching defect, less pigmentation, awkward position, and heart edema of the developing zebrafish embryos treated with six different concentrations of 100% *v/v* methanol extract of *P. malayana* leaves.

100% Methanol Extract (µg/mL)	Eye Size × 10^4^ (µm^2^) 96 hpf	Yolk Size × 10^5^ (µm^2^) 24 hpf	Body Length (mm) 96 hpf	Hatching Defect	Less Pigmentation	Yolk Edema	Crooked Backbone	Heart Edema
6.25	6.1 ± 0.3 ^a^	2.5 ± 0.2 ^b^	2.5 ± 0.1 ^b^	+	+	−	+	+
3.125	5.6 ± 0.5 ^b^	2.6 ± 0.1 ^b^	2.5 ± 0.2 ^b^	−	−	−	−	−
NC	5.7 ± 0.3 ^b^	2.6 ± 0.2 ^b^	2.8 ± 0.2 ^a^	−	−	−	−	−
SC	5.7 ± 0.3 ^a,b^	2.6 ± 0.2 ^b^	2.8 ± 0.2 ^a^	−	−	−	−	−
PC	2.4 ± 0.7 ^c^	3.2 ± 0.3 ^a^	NA	+	NA	+	NA	+

(−): not present; (+): present; NA: not analyzed due to hatching defect; NC: negative control; SC: solvent control; PC: positive control. The values are presented as mean ± SD, *n* = 20. Different letters indicate significant differences (*p* < 0.05).

**Table 6:** Putative compounds identified in the methanol and water extracts of *P. malayana* leaves by GC-MS after derivatization.

Extracts	Putative Compounds	Retention Time (min)	% of Area	Similarity Index	Molecular Formula
Methanol	Phosphonic acid	16.261	0.03	93	H_2_O_3_P^+^
	Resorcinol	19.941	0.34	95	C_6_H_6_O_2_
	1,3,5-benzenetriol	37.213	0.61	98	C_6_H_6_O_3_
	DL-arabinose	49.569	0.81	93	C_5_H_10_O_5_
	D-ribose	50.112	3.86	93	C_5_H_10_O_5_
	D-mannose	50.815	2.87	91	C_6_H_12_O_6_
	D-psicose	51.393	0.12	90	C_6_H_12_O_6_
	L-sorbose	52.941	1.47	91	C_6_H_12_O_6_
	Palmitic acid	58.142	0.04	98	C_16_H_32_O_2_
	Myo-inositol	60.234	0.15	94	C_6_H_12_O_6_
	1-monopalmitin	81.935	0.17	93	C_19_H_38_O_4_
	Sucrose	83.878	4.21	93	C_12_H_22_O_11_
	Beta-tocopherol	100.870	0.10	93	C_28_H_48_O_2_
	Alpha-tocopherol	101.104	0.24	98	C_29_H_50_O_2_
	24-epicampesterol	104.362	0.03	90	C_28_H_48_O
	Stigmast-5-ene	107.083	0.31	99	C_29_H_50_
Water	Resorcinol	19.941	0.21	91	C_6_H_6_O_2_
	Hydroquinone	21.690	0.42	94	C_6_H_6_O_2_
	D-ribino-1,4-lactone	36.384	0.11	97	C_5_H_8_O_5_
	1,3,5-benezenetriol	37.213	0.45	98	C_6_H_6_O_3_

**Table 7:** 2-NBDG treatment groups.

Groups	2-NBDG (µg/mL)	Insulin (µg/mL)	Rosiglitazone (µM)	Methanol Extract (µg/mL)	Water Extract (µg/mL)
NC	100	-	-	-	-
IC	100	10	-	-	-
PC	100	10	10	-	-
Insulin-sensitizing activity of methanol extract	100	10	-	5, 10, 15	-
Insulin-sensitizing activity of water extract	100	10	-	-	5, 10, 15

NC = Negative control; IC = Insulin control; PC = Positive control.

## Data Availability

Data is contained within the article.

## Supplementary Materials

The following are available online at https://www.mdpi.com/article/10.3390/plants10122688/s1, Figure S1: Comparison between the fragment *m*/*z* spectrum of phosphonic acid from the sample and NIST database (SI = 98%), Figure S2: Comparison between the fragment *m*/*z* spectrum of resocinol from the sample and NIST database (SI = 95%), Figure S3: Comparison between the fragment *m*/*z* spectrum of 1,3,5-benzenetriol from the sample and NIST database (SI = 98%), Figure S4: Comparison between the fragment *m*/*z* spectrum of Dl-arabinose from the sample and NIST database (SI = 93%), Figure S5: Comparison between the fragment *m*/*z* spectrum of D-ribose from the sample and NIST database (SI = 93%), Figure S6: Comparison between the fragment *m*/*z* spectrum of D-mannose from the sample and NIST database (SI = 91%), Figure S7: Comparison between the fragment *m*/*z* spectrum of D-psicose from the sample and NIST database (SI = 90%), Figure S8: Comparison between the fragment *m*/*z* spectrum of L-sorbose from the sample and NIST database (SI = 91%), Figure S9: Comparison between the fragment *m*/*z* spectrum of palmitic acid from the sample and NIST database (SI = 98%), Figure S10: Comparison between the fragment *m*/*z* spectrum of myo-inositol from the sample and NIST database (SI = 94%), Figure S11: Comparison between the fragment *m*/*z* spectrum of 1-monopalmitin from the sample and NIST database (SI = 93%), Figure S12: Comparison between the fragment *m*/*z* spectrum of sucrose from the sample and NIST database (SI = 93%), Figure S13: Comparison between the fragment *m*/*z* spectrum of beta-tocopherol from the sample and NIST database (SI = 93%), Figure S14: Comparison between the fragment *m*/*z* spectrum of alpha-tocopherol from the sample and NIST database (SI = 98%), Figure S15: Comparison between the fragment *m*/*z* spectrum of 24-epicampesterol from the sample and NIST database (SI = 90%), Figure S16: Comparison between the fragment *m*/*z* spectrum of stigmast-5-ene from the sample and NIST database (SI = 99%), Figure S17: Comparison between the fragment *m*/*z* spectrum of hydroquinone from the sample and NIST database (SI = 94%), Figure S18: Comparison between the fragment *m*/*z* spectrum of D-ribino-1,4-lactone from the sample and NIST database (SI = 97%).
