# Supplementary figures and images for: Preliminary Phytochemical Screening, In Vitro Antidiabetic, Antioxidant Activities, and Toxicity of Leaf Extracts of Psychotria malayana Jack
Source: Plants (Basel). 2021 Dec 7;10(12):2688. doi: 10.3390/plants10122688 (PMC8707723; doi:10.3390/plants10122688)

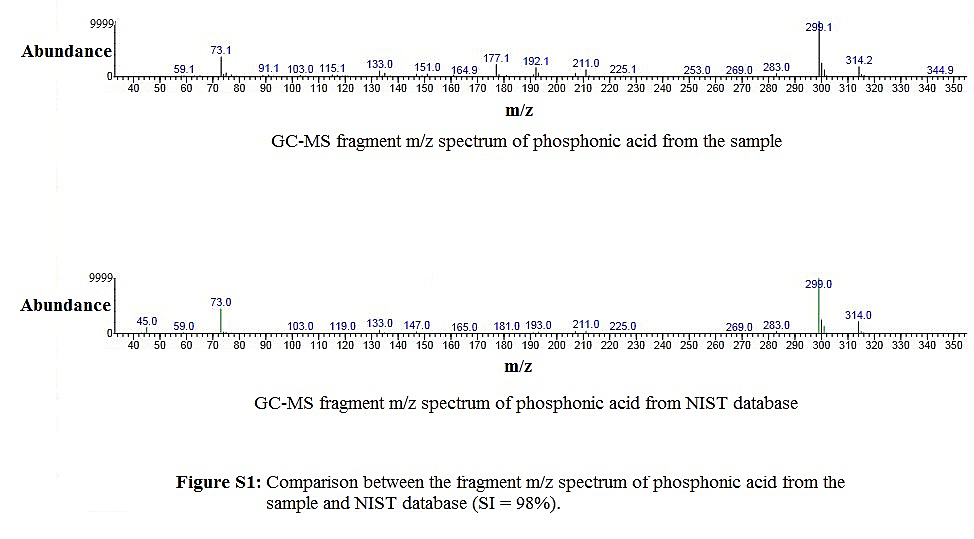

Supplement: Supplementary file 1 [file plants-10-02688-s001.zip › All Supplementary Figures (S1-S18)/All Supplementary Figures (S1-S18)/Figure S1 (Phosphonic acid).tiff]

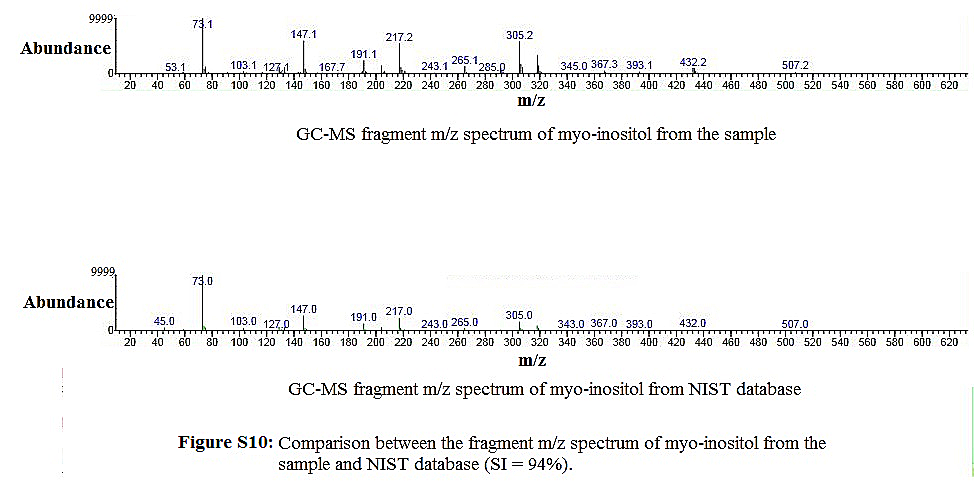

Supplement: Supplementary file 1 [file plants-10-02688-s001.zip › All Supplementary Figures (S1-S18)/All Supplementary Figures (S1-S18)/Figure S10 (Myo-unisitol).tiff]

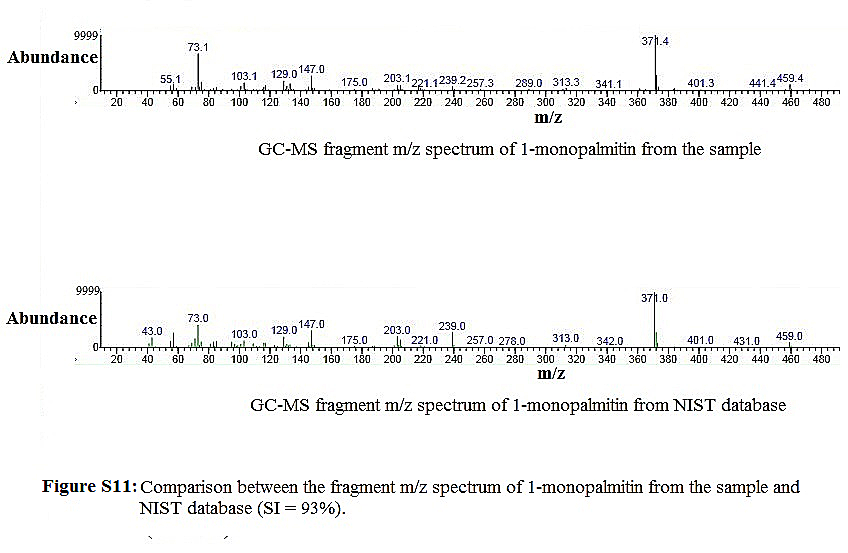

Supplement: Supplementary file 1 [file plants-10-02688-s001.zip › All Supplementary Figures (S1-S18)/All Supplementary Figures (S1-S18)/Figure S11 (1-monopalmitin).tiff]

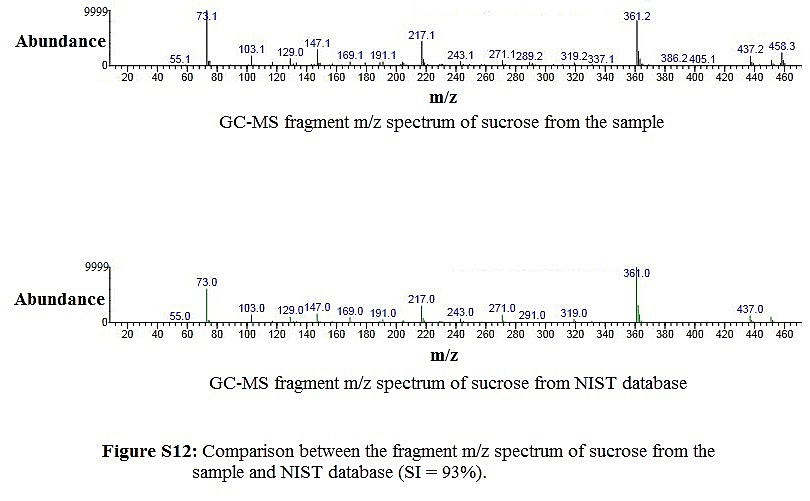

Supplement: Supplementary file 1 [file plants-10-02688-s001.zip › All Supplementary Figures (S1-S18)/All Supplementary Figures (S1-S18)/Figure S12 (sucrose).tiff]

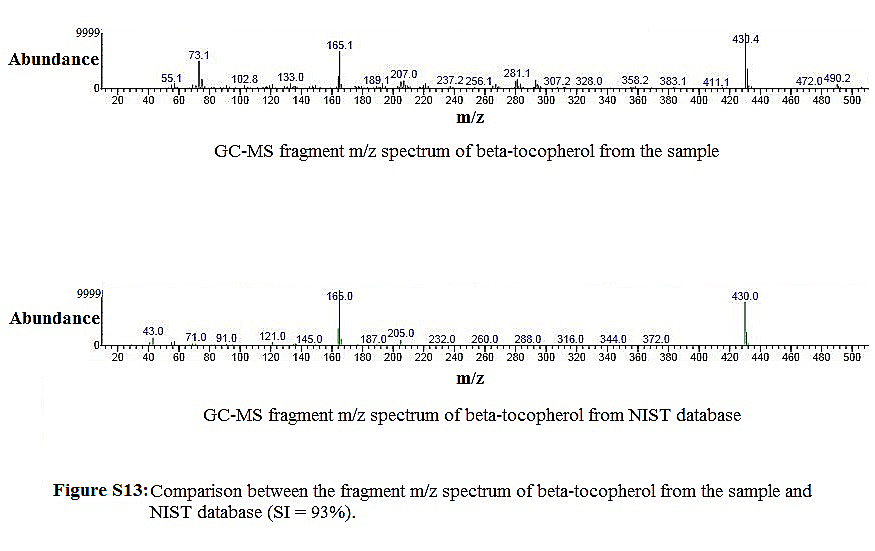

Supplement: Supplementary file 1 [file plants-10-02688-s001.zip › All Supplementary Figures (S1-S18)/All Supplementary Figures (S1-S18)/Figure S13 (Beta-tocopherol).tiff]

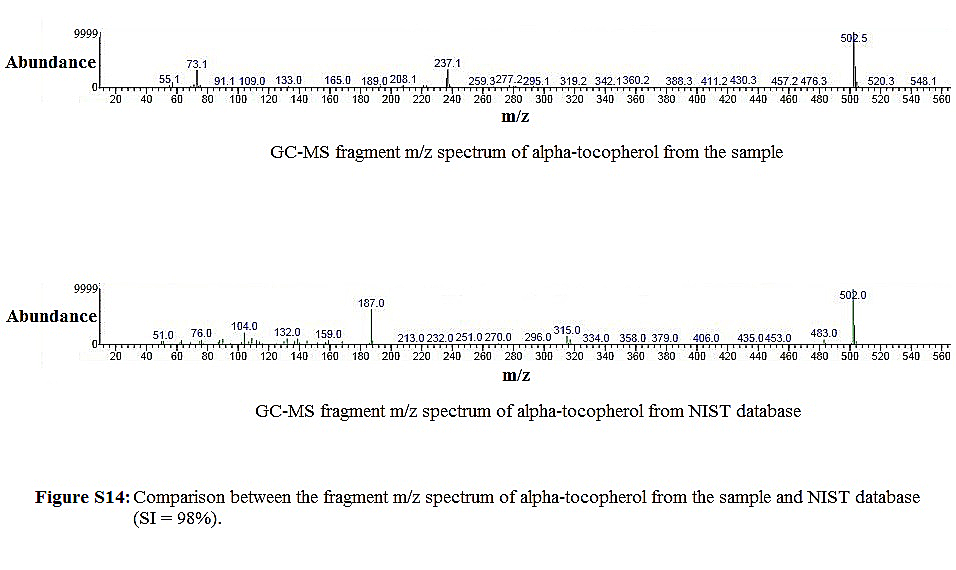

Supplement: Supplementary file 1 [file plants-10-02688-s001.zip › All Supplementary Figures (S1-S18)/All Supplementary Figures (S1-S18)/Figure S14 (Alpha-tocopherol).tiff]

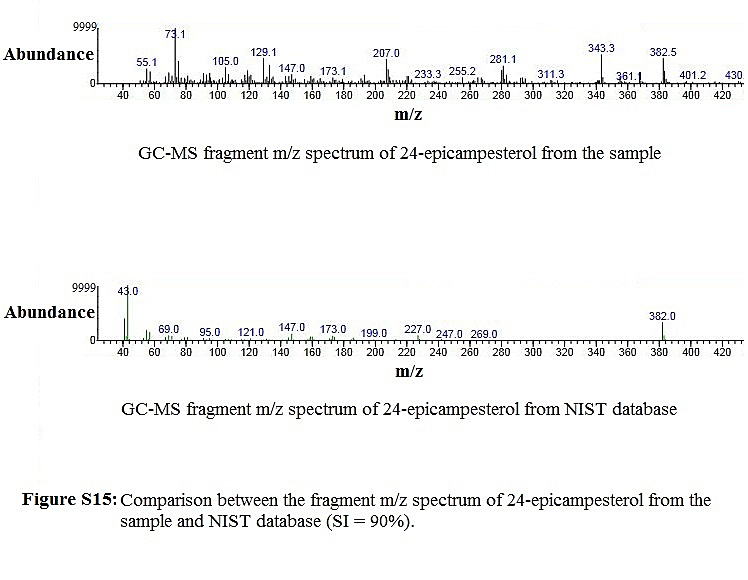

Supplement: Supplementary file 1 [file plants-10-02688-s001.zip › All Supplementary Figures (S1-S18)/All Supplementary Figures (S1-S18)/Figure S15 (24-epicampesterol).tiff]

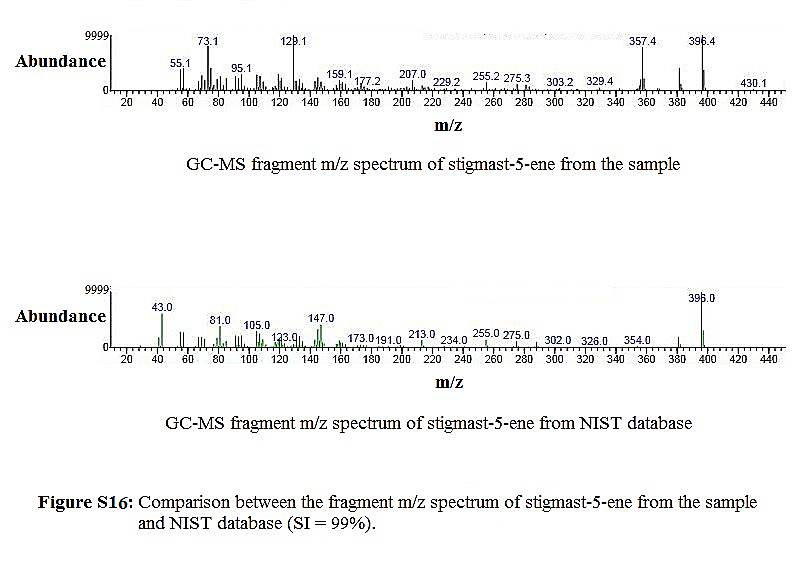

Supplement: Supplementary file 1 [file plants-10-02688-s001.zip › All Supplementary Figures (S1-S18)/All Supplementary Figures (S1-S18)/Figure S16 (stigmast-5-ene).tiff]

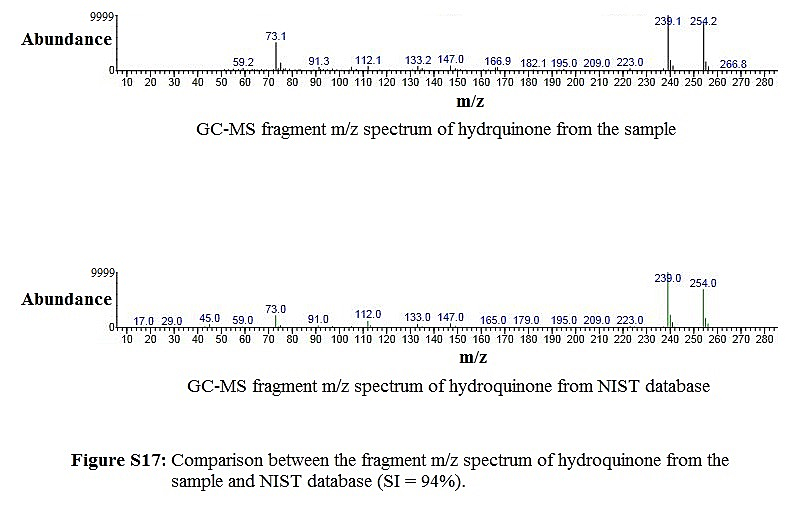

Supplement: Supplementary file 1 [file plants-10-02688-s001.zip › All Supplementary Figures (S1-S18)/All Supplementary Figures (S1-S18)/Figure S17 (Hydroquinone).tiff]

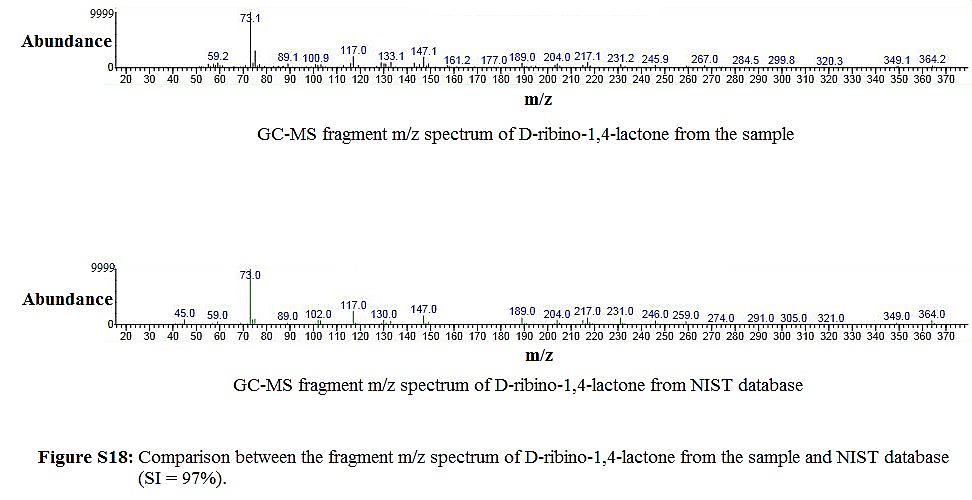

Supplement: Supplementary file 1 [file plants-10-02688-s001.zip › All Supplementary Figures (S1-S18)/All Supplementary Figures (S1-S18)/Figure S18 (D-Ribino-1,4-lactone).tiff]

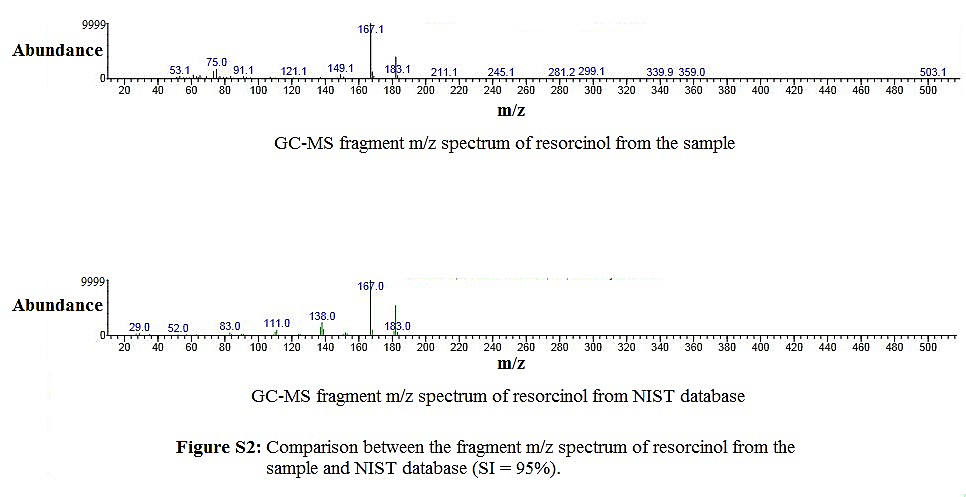

Supplement: Supplementary file 1 [file plants-10-02688-s001.zip › All Supplementary Figures (S1-S18)/All Supplementary Figures (S1-S18)/Figure S2 (Resorcinol).tiff]

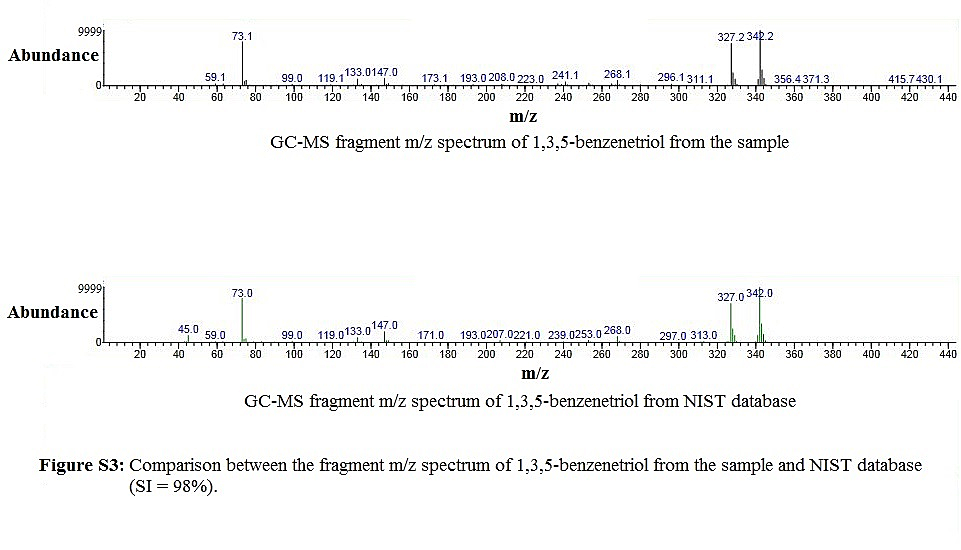

Supplement: Supplementary file 1 [file plants-10-02688-s001.zip › All Supplementary Figures (S1-S18)/All Supplementary Figures (S1-S18)/Figure S3 (1,3,5-benzenetriol).tiff]

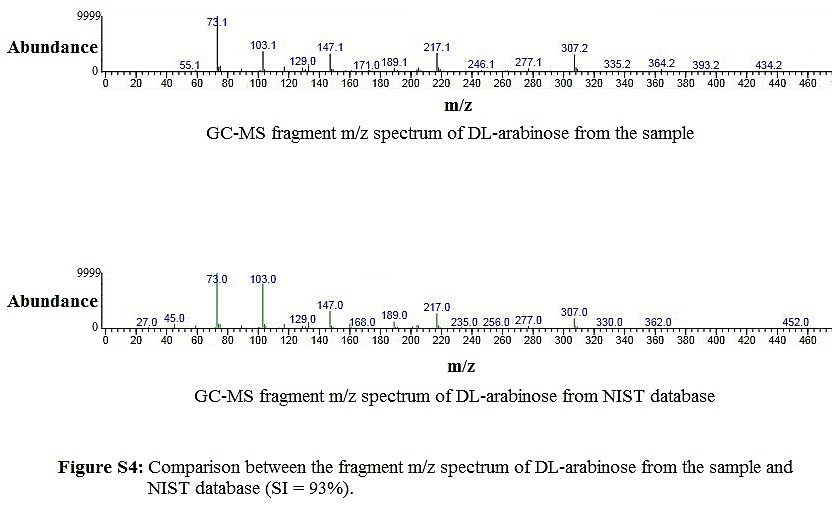

Supplement: Supplementary file 1 [file plants-10-02688-s001.zip › All Supplementary Figures (S1-S18)/All Supplementary Figures (S1-S18)/Figure S4 (DL-arabinose).tiff]

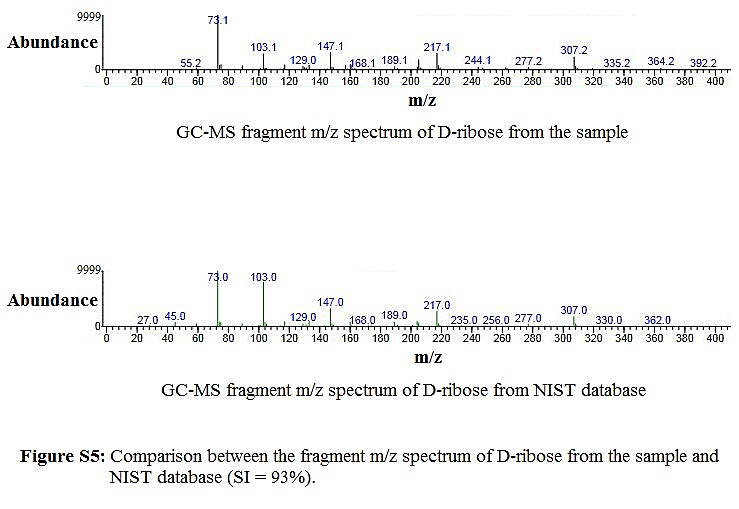

Supplement: Supplementary file 1 [file plants-10-02688-s001.zip › All Supplementary Figures (S1-S18)/All Supplementary Figures (S1-S18)/Figure S5 (D-ribose).tiff]

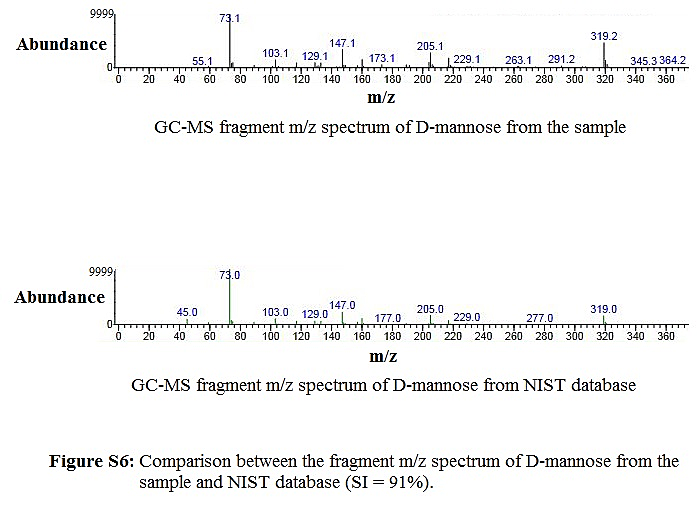

Supplement: Supplementary file 1 [file plants-10-02688-s001.zip › All Supplementary Figures (S1-S18)/All Supplementary Figures (S1-S18)/Figure S6 (D-mannose).tiff]

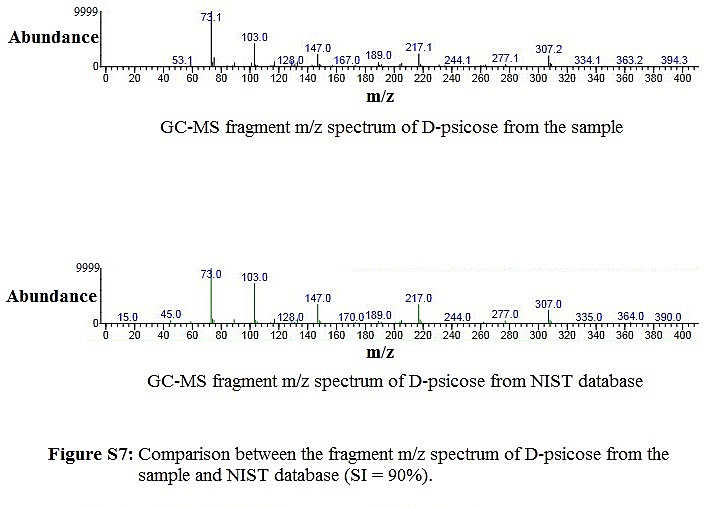

Supplement: Supplementary file 1 [file plants-10-02688-s001.zip › All Supplementary Figures (S1-S18)/All Supplementary Figures (S1-S18)/Figure S7 (D-psicose).tiff]

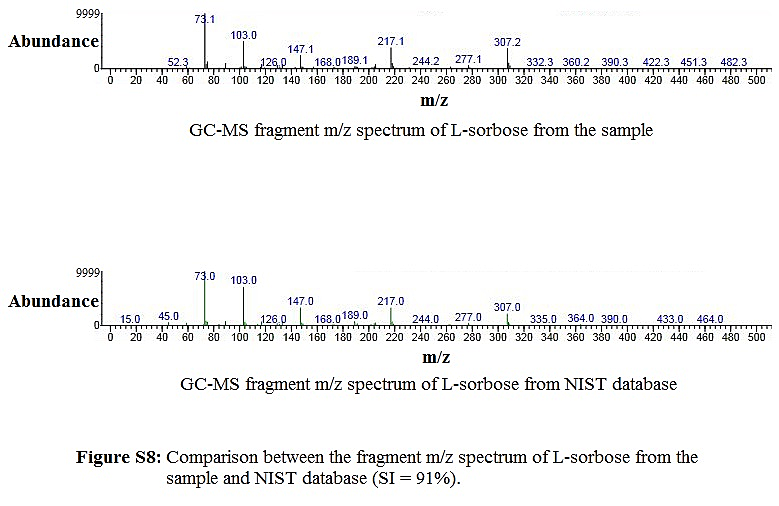

Supplement: Supplementary file 1 [file plants-10-02688-s001.zip › All Supplementary Figures (S1-S18)/All Supplementary Figures (S1-S18)/Figure S8 (L-sorbose).tiff]

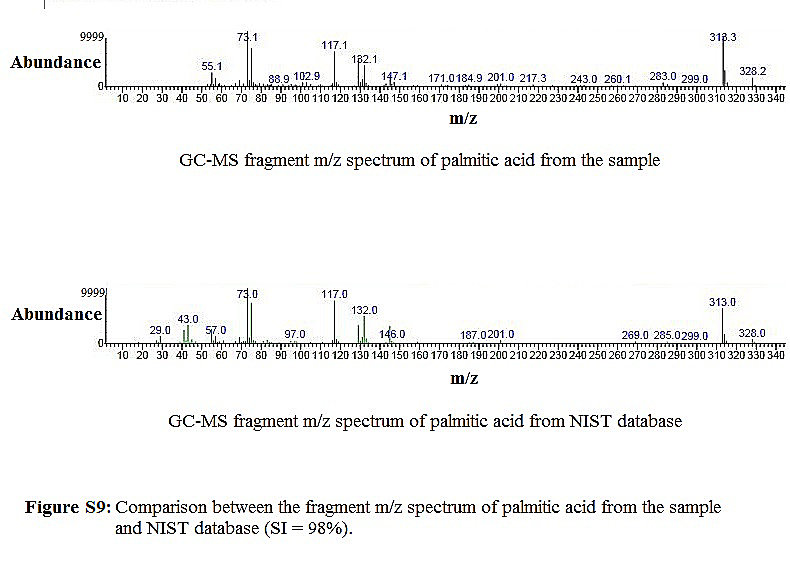

Supplement: Supplementary file 1 [file plants-10-02688-s001.zip › All Supplementary Figures (S1-S18)/All Supplementary Figures (S1-S18)/Figure S9 (Palmitic acid).tiff]
